# PIKfyve regulates melanosome biogenesis

**DOI:** 10.1371/journal.pgen.1007290

**Published:** 2018-03-27

**Authors:** Marc C. Liggins, Jessica L. Flesher, Sohail Jahid, Priya Vasudeva, Victoria Eby, Shunsuke Takasuga, Junko Sasaki, Takehiko Sasaki, Raymond E. Boissy, Anand K. Ganesan

**Affiliations:** 1 Department of Dermatology, University of California, San Diego, San Diego, CA, United States of America; 2 Department of Biological Chemistry, University of California, Irvine, Irvine, CA, United States of America; 3 Department of Dermatology, University of California, Irvine, Irvine, CA, United States of America; 4 Department of Dermatology, University of Cincinnati, Cincinnati, OH, United States of America; 5 Department of Medical Biology, Akita University School of Medicine, Akita, Japan; University of Wisconsin, UNITED STATES

## Abstract

PIKfyve, VAC14, and FIG4 form a complex that catalyzes the production of PI(3,5)P_2_, a signaling lipid implicated in process ranging from lysosome maturation to neurodegeneration. While previous studies have identified VAC14 and FIG4 mutations that lead to both neurodegeneration and coat color defects, how PIKfyve regulates melanogenesis is unknown. In this study, we sought to better understand the role of PIKfyve in melanosome biogenesis. Melanocyte-specific PIKfyve knockout mice exhibit greying of the mouse coat and the accumulation of single membrane vesicle structures in melanocytes resembling multivesicular endosomes. PIKfyve inhibition blocks melanosome maturation, the processing of the melanosome protein PMEL, and the trafficking of the melanosome protein TYRP1. Taken together, these studies identify a novel role for PIKfyve in controlling the delivery of proteins from the endosomal compartment to the melanosome, a role that is distinct from the role of PIKfyve in the reformation of lysosomes from endolysosomes.

## Author summary

In order for a cell to develop and maintain functional organelles, proteins must be delivered to developing organelles in a precise, sequential fashion. In this study, we used a well-established model for organelle biogenesis (the melanosome) to understand how phosphoinositides regulate vesicle trafficking and melanogenesis. Blocking PI(3,5)P_2_ synthesis inhibits melanosome maturation and induces the accumulation of multivesicular endosomes. Intriguingly, this role is different from the role of PI(3,5)P_2_ in lysosomal reformation. Taken together, these studies identify distinct roles for PI(3,5)P_2_ in melanosome biogenesis.

## Introduction

Melanin, a pigment produced within uveal and epidermal melanocytes, absorbs UV radiation, protecting the eyes and skin from UV-induced DNA damage [1]. Melanin is synthesized in a lysosome-related organelle called the melanosome, which develops through four distinct stages that are readily distinguishable by electron microscopy [2, 3]. Several human monoallelic disorders that present with hypopigmentation also have deficits in the biogenesis of lysosomes and lysosome-related organelles [4], highlighting the utility of melanosome biogenesis as a model system to define pathways that regulate organelle biogenesis [3, 5].

The complex process of melanosome biogenesis initiates when specialized early endosomes bud off into spherical vacuoles known as stage I melanosomes, marked by the presence of melanoma antigen recognized by T cells 1 (MART-1) and the premelanosome protein (PMEL) [6–8]. Cleavage and fibrillation of PMEL marks the transition to stage II melanosomes [7] where PMEL fibrils serve as scaffold for melanin polymerization and deposition [9]. Tyrosinase (TYR), and tyrosinase-related proteins 1 and 2 (TYRP1 & TYRP2) are three key enzymes involved in producing melanin [10]. These proteins are glycosylated within the Golgi and packaged into adaptor protein-3 (AP-3) or -1 (AP-1) clathrin coated transport vesicles, which are transported to and fuse with stage II melanosomes [11]. The initiation of tyrosinase enzymatic activity allows for the production of melanin [10], which is then deposited onto PMEL fibrils in stage III melanosomes [9]. Mature, stage IV melanosomes are highly pigmented vesicles, which are opaque structures on electron microscopy filled with electron dense melanin [3, 5]. Finally, mature melanosomes are transferred to neighboring keratinocytes [12, 13] through a process that is poorly understood.

Numerous studies have determined that proteins are delivered to the developing melanosome through multivesicular endosomes (MVEs). The stage I melanosome protein MART-1 is modified by K63 ubiquitin chains and delivered to a MVE [14]. This protein is ultimately transported to the stage I melanosome by a mechanism requiring the ESCRT (endosomal sorting complex required for transport) machinery [14]. In contrast, PMEL is sorted to intraluminal vesicles of MVEs by a process that is independent of ESCRT [15], and are then cleaved into PMEL fibrils in the developing melanosome [9]. TYRP1 is trafficked to the melanosome limiting membrane through a process that requires both ESCRT I [16] and AP-1 [17]. TYR enters the MVE and then is rapidly re-recruited by a complex containing AP-3 into vesicles that ultimately transports this cargo to Stage II melanosomes [18]. While it is clear that many proteins are trafficked through multivesicular endosomes en route to melanosomes, it is still not known what signals are required for these proteins to exit the MVEs or what signals regulate the fusion of MVE-derived transport vesicles with the melanosome.

Phosphoinositides (PI) have been implicated in controlling the fusion of transport vesicles with its desired target membrane [19, 20]. The low abundance lipid PI(3,5)P_2_ has undefined roles in organelle biogenesis but has been implicated in physiologic processes including autophagy [21–23], lysosome biogenesis [24–29], cytokine production [30], and vesicular trafficking [28, 31]. PI(3,5)P_2_ is synthesized by the mammalian PI5-kinase core complex, composed of the kinase PIKfyve, FIG4, and a VAC14 dimer [32–34]. Intriguingly, FIG4 and VAC14 mutant mice exhibit coat color defects and vesicle trafficking defects in the central nervous system (CNS), and the CNS effects are related to an accumulation of autophagosomes [35]. PIKfyve inhibition *in vitro* has been recently shown to inhibit the reformation of lysosomes from endolysosomes [36]. In summary, these studies implicate functional roles for the PIKfyve protein and the lipid it produces (PI(3,5)P_2_) in autophagy and lysosomal biogenesis/turnover.

In this study, we sought to better understand how PIKfyve regulates melanosome biogenesis. We determine that loss or inhibition of PIKfyve in melanocytes blocks melanosome maturation, resulting in the accumulation of single membrane vesicle structures that contain intraluminal vesicles. Taken together, these studies define a specific role for phosphoinositides in regulating the delivery of proteins from multivesicular endosomes to melanosomes, phenotypes that are distinct from the role of PIKfyve in lysosome biogenesis and turnover.

## Results

### Loss of PIKfyve leads to pigment loss *in vivo*

**Fig 1:**
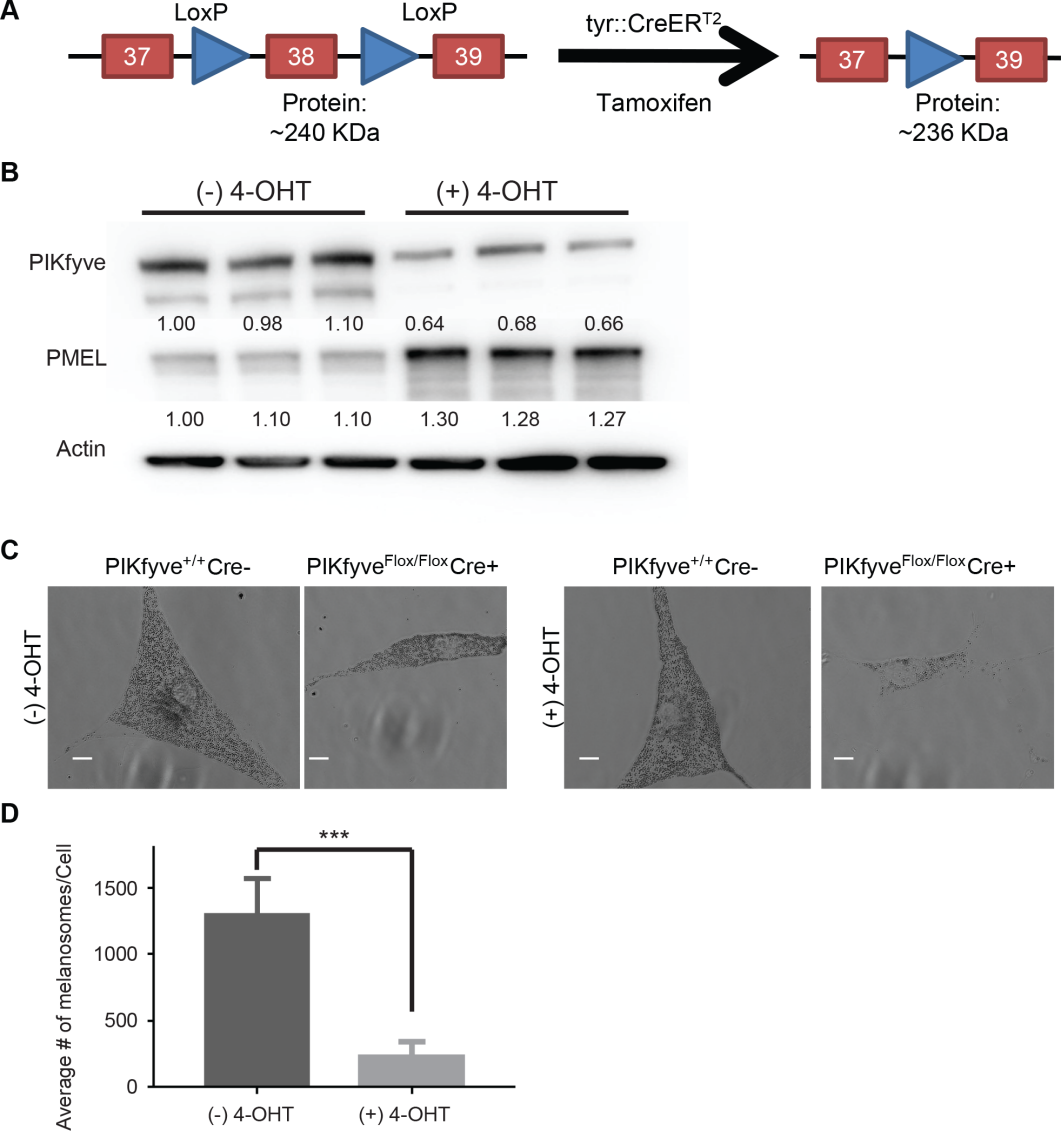
Loss of PIKfyve alters melanosome number. A) Schematic of *Tyrosinase*::*Cre*^*ERT2*^ *PIKfyve*^*Flox/Flox*^ knockout mice. In this model, the *PIKfyve* exon 38 (kinase domain) is flanked by intronic LoxP sites. Cells were treated with 4-hydroxytamoxifen (4-OHT) and mice were administered feed containing tamoxifen (TAM) to remove exon 38 resulting in the inactivation of the PIKfyve kinase. B) Six different batches of primary melanocytes isolated from neonatal *Tyrosinase*::*Cre*^*ERT2*^; *PIKfyve*^*Flox/Flox*^ were treated with 4-OHT or vehicle control for 48hrs. Protein lysates were collected and immunoblotted with the indicated Abs to measure PIKfyve and Pmel levels. C) Cells from *Tyrosinase*::*Cre*^*ERT2*^; *PIKfyve*^*Flox/Flox*^ or wild type mice treated with 4-OHT or vehicle were then fixed and imaged using phase contrast microscopy, scale bar = 10μm. D) The number of melanosomes per cell in *Tyrosinase*::*Cre*^*ERT2*^; *PIKfyve*^*Flox/Flox*^ melanocytes treated with 4-OHT or vehicle was counted and quantified using ImageJ. ***, p < 0.001 using a two-tailed Student’s paired T test.

PIKfyve forms a complex with VAC14 and FIG4 [32, 37] which then phosphorylates PI(3)P to PI(3,5)P_2_ [38] and PIP to PI(5)P [39, 40]. VAC14 and FIG4 mutants are characterized by early lethality and accumulation of vacuoles in the CNS with accompanying coat color defects [37, 41, 42]. PIKfyve knockout mice die during embryonic development [43], making it difficult to assess the effects of PIKfyve on melanogenesis. To better elucidate the role of PIKfyve in melanosome biogenesis, we generated melanocyte-specific, inducible PIKfyve knockout mice by crossing an established *PIKfyve*^*Flox/Flox*^ strain [44] with an established melanocyte-specific, inducible Cre strain under a tyrosinase promoter [45] on a pure C57B6 background. Induction of Cre recombination with tamoxifen results in the excision of the kinase domain of PIKfyve producing a slightly smaller protein than the full length PIKfyve (Fig 1A). Previously published studies suggested that truncated PIKfyve is unstable- infecting *PIKfyve*^*Flox/Flox*^ mouse embryonic fibroblasts with adenovirus expressing Cre recombinase resulted in the loss PIKfyve protein [44]. Initial experiments verified that treating *TyrCreER*^*T2*^ *PIKfyve*^*Flox/Flox*^ melanocytes with tamoxifen also inhibited the accumulation of PIKfyve protein (Fig 1B). Notably, tamoxifen treatment did not result in the complete deletion of PIKfyve as was described previously [44], indicating that Cre recombination in this model system is less efficient. In addition, PIKfyve deletion inhibited the accumulation of pigmented melanosomes (Fig 1C and 1D), while also resulting in the accumulation of full length PMEL protein (Fig 1B).

**Fig 2:**
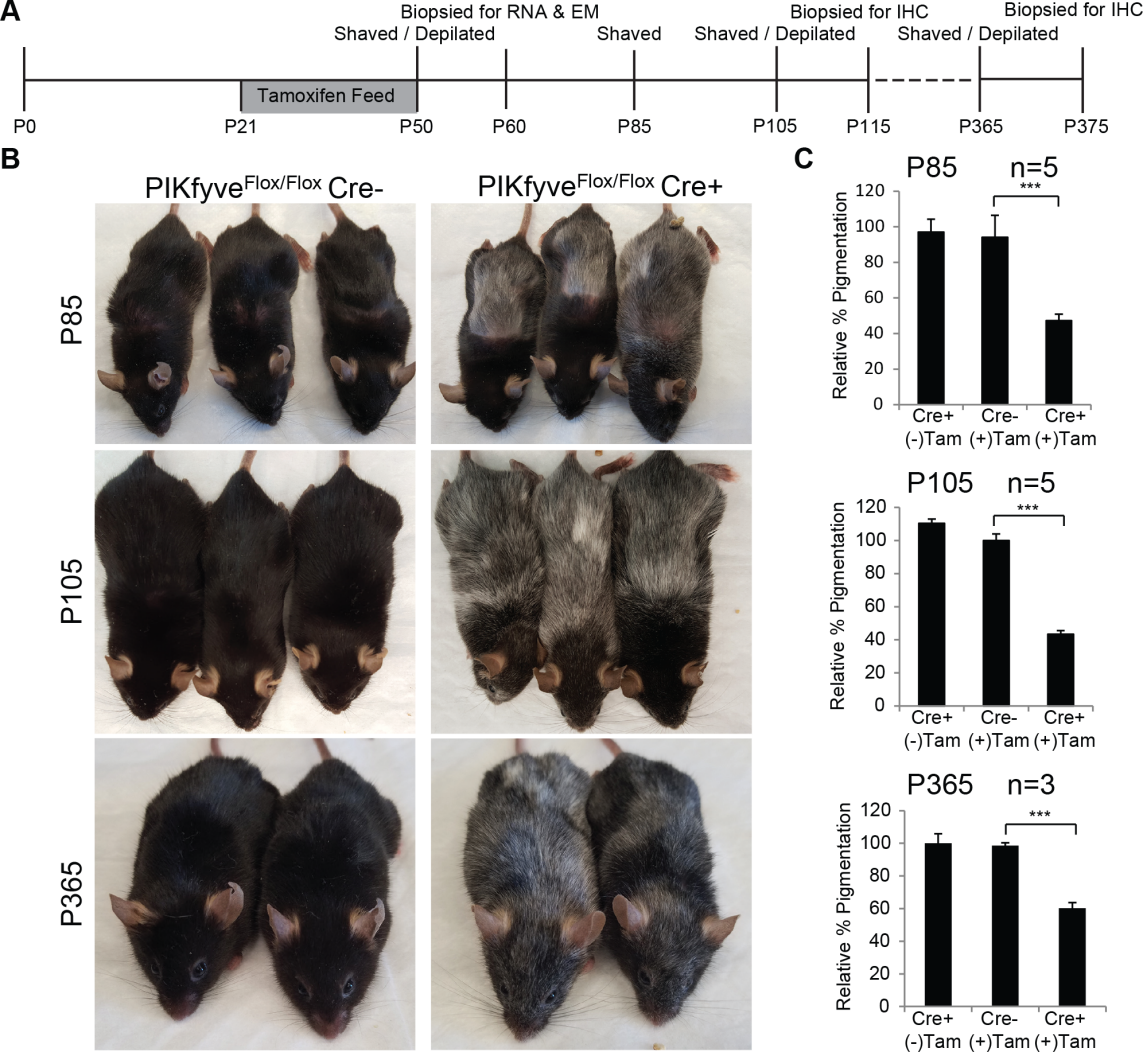
Melanocyte specific PIKfyve knockout mice exhibit hair greying. A) Timeline of *in vivo* experiments. Five *Tyrosinase*::*Cre*^*ERT2*^; *PIKfyve*^*Flox/Flox*^ and 5 *PIKfyve*^*Flox/Flox*^ mice were administered tamoxifen chow for 29 days. A control group of 5 *Tyrosinase*::*Cre*^*ERT2*^; *PIKfyve*^*Flox/Flox*^ mice were fed a control diet throughout the course of the experiment. All mice were shave depilated at p50 and subsequently fed normal chow beginning at p50 for the subsequent days. Gray bar denotes the duration that mice, with the exception of the control group, were on tamoxifen feed. B) Littermates were photographed at P85, P105, and P365. C) Mouse hair from the genotypes indicated was dissolved in solune-350 and melanin quantitation was performed as described. The relative amount of melanin in the hair was calculated relative to *Cre*- controls at P85, P105,and P365. Data shown are mean ± S.D. (n = 5 or 3 as indicated by error bars. ***, p < 0.001 using a two-tailed Student’s paired T test.

To examine the effect of PIKfyve knockout on melanogenesis, *TyrCreER*^*T2*^ *PIKfyve*^*Flox/Flox*^ mice were administered tamoxifen-containing chow for 29 days beginning at P21 (Fig 2A) to induce Cre-mediated excision of the kinase domain of PIKfyve. Mice were photographed initially (S1A Fig), shaved and depilated on a region of their back at P50, and hairs were allowed to regrow. *TyrCreER*^*T2*^ *PIKfyve*^*Flox/Flox*^ mice fed tamoxifen for 29 days accumulated numerous white hairs that initially presented in the shaved and depilated area and were visually apparent at P85 (Figs 2B and S1B). The same hair phenotype was not observed in Cre negative *PIKfyve*^*Flox/Flox*^ mice fed tamoxifen for 29 days or *TyrCreER*^*T2*^ *PIKfyve*^*Flox/Flox*^ mice that were administered a normal diet (Figs 2B, S1B and S1C). Additional experiments revealed that the weights of *PIKfyve*^*Flox/Flox*^ mice that were fed tamoxifen were similar regardless of whether they expressed *TyrCreER*^*T2*^ (S1D Fig), indicating that the smaller relative size of the *TyrCreER*^*T2*^ *PIKfyve*^*Flox/Flox*^ mice fed tamoxifen chow as compared to mice fed normal chow was a consequence of diet and not PIKfyve loss. Hair from the backs of experimental mice was solubilized and the relative accumulation of melanin was quantified using standard spectrophotometric methods [46, 47]. Hairs from the *TyrCreER*^*T2*^ *PIKfyve*^*Flox/Flox*^ mice that were fed tamoxifen accumulated 50% less melanin as compared to mice that were not fed tamoxifen or Cre negative controls (Fig 2C).

To better assess whether this phenotype was exclusively related to an effect on melanogenesis, we allowed the hairs to regrow after shave depilation and observed whether there was an increased accumulation of white hair after the mice were switched off tamoxifen feed. More white hairs were visually apparent after the mice were fed a normal diet for an additional 20 days (p105, Figs 2B, S1B and S1C). This phenotype appeared to be progressive initially as even less melanin accumulated in the hair of *PIKfyve*^*Flox/Flox*^ mice after they were taken off tamoxifen chow (Fig 2B). Over the course of a year, *PIKfyve*^*Flox/Flox*^ mice continued to accumulate numerous white hairs on the head and upper back, areas that had never been shave depilated (S1B and S1C Fig), indicating that the phenotype was accelerated but not induced by shave depilation. The observed phenotype did not progress completely, as the mice continued to maintain the same relative level of depigmentation over the course of a year (S1C Fig). Taken together, these results indicate that PIKfyve loss inhibits melanin accumulation in the mouse hair and this phenotype is not progressive as would be expected if the phenotype were related to stem cell depletion [48].

**Fig 3:**
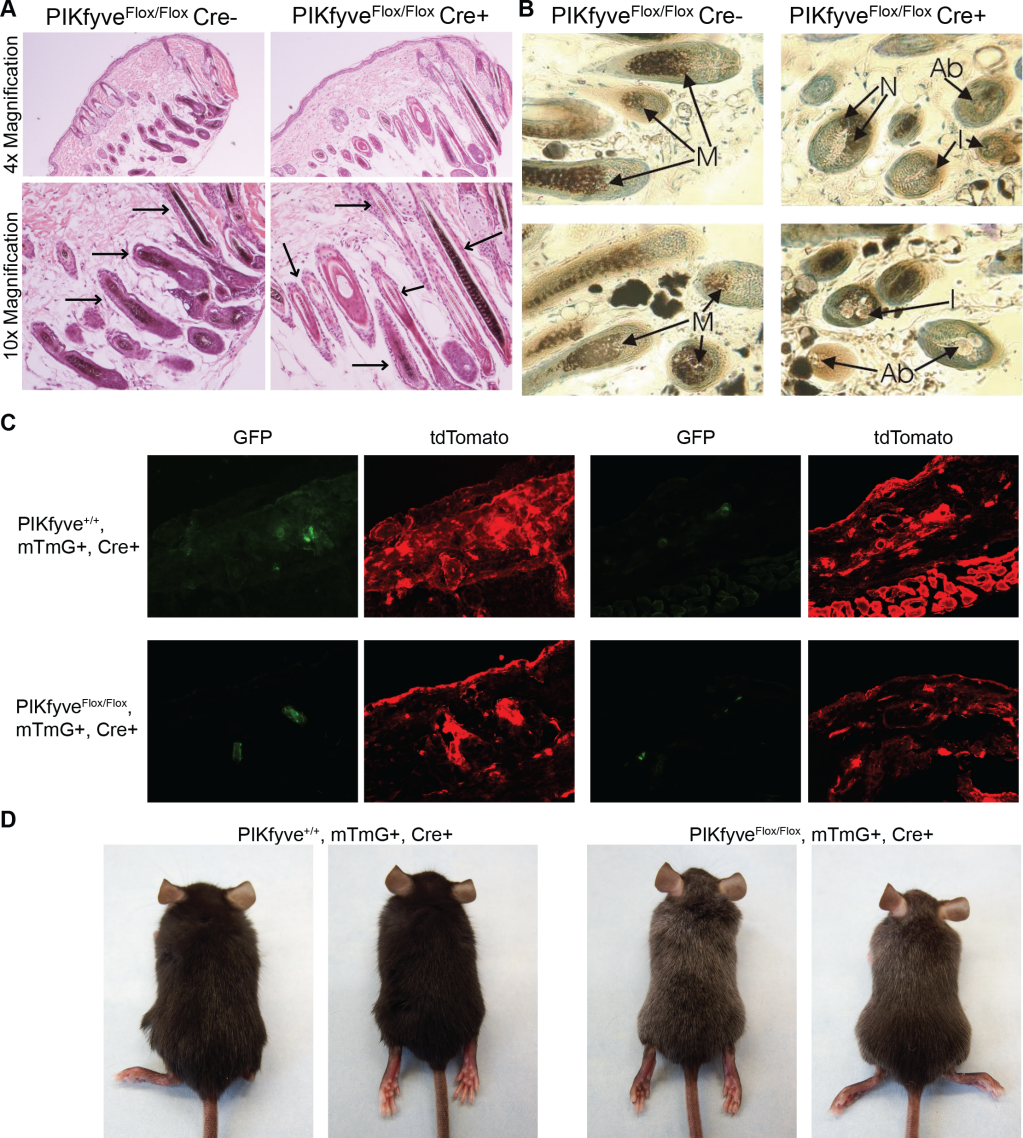
Melanocyte specific PIKfyve knockout mice exhibit vacuolar accumulation in select mouse hairs. A) Haematoxylin and eosin stained sections from mice of the indicated genotypes at p60 was examined by light microscopy at 4x and 10x magnification. B) Toluene Blue stained semi-thin sections from mice of the indicated genotypes examined by light microscopy at 100x magnification. Anagen hair follicles from control PIKfyve^Flox/Flox^Cre- mice (left panel) demonstrating normal melanocytes (M) at the epidermal/dermal papilla interface and from PIKfyve^Flox/Flox^Cre+ mice (right panel) demonstrating melanocytes that appear morphologically normal (N), abnormal without containing melanin (Ab) and intermediate with minimal amount of melanin (I). C) Skin from *TyrCreER*^*T2*^ *PIKfyve*^*Flox/Flox*^ *ROSA*^*mTmG/+*^ at P100 was embedded in OCT and examined by fluorescent microscopy at 4x magnification. D) Representative images of *TyrCreER*^*T2*^ *PIKfyve*^*Flox/Flox*^ *ROSA*^*mTmG/+*^ mice and *TyrCreER*^*T2*^ *PIKfyve*^*++*^ *ROSA*^*mTmG/+*^ mice at P50. Note the early greying phenotype shown here is similar to what is observed in *TyrCreER*^*T2*^ *PIKfyve*^*Flox/Flox*^ mice at p50.

To better understand how PIKfyve loss influences melanin accumulation, we next sought to characterize the PIKfyve knockout mice at the cellular level. Initial studies sought to better understand why *TyrCreER*^*T2*^ *PIKfyve*^*Flox/Flox*^ mice fed tamoxifen were not completely white but instead accumulated sporadic white hairs. *TyrCreER*^*T2*^ *PIKfyve*^*Flox/Flox*^ littermates fed either a control diet or tamoxifen diet were shave depilated at P50. In both groups, mice had entered the 3^rd^ anagen by P60 with visible hair regrowth (S2A Fig). Histology on skin collected at P60 revealed that mice fed tamoxifen had both pigmented and unpigmented hair growing in follicles while mice fed the control diet had fully pigmented hair growth (S2B Fig). Since pigmented and unpigmented hairs are present in neighboring follicles, this suggests that loss of PIKfyve does not affect the growth of the hair. Further studies examined the structure of the hairs histologically and verified that the hairs from PIKfyve knockout mice had the same morphology as wild-type mice with the exception that a number of the hairs in the knockout mouse lacked pigment (Fig 3A). High magnification images of these hair follicles revealed that some of them exhibited the accumulation of cells with intracellular vesicles with some residual pigment (Fig 3B, I = intermediate), while other hairs exhibited the accumulation of cells with intracellular vesicles with little to no pigment (Fig 3B, Ab = abnormal). Still other hair follicles appeared normal (Fig 3B, N = Normal). The observation that not all hair follicles in the mice exhibit a vacuolated phenotype is consistent with *in vitro* results indicating that deletion of PIKfyve was not 100% efficient (Fig 1B). To further verify that the observed phenotypes were not a consequence of stem cell loss, *TyrCreER*^*T2*^ *PIKfyve*^*Flox/Flox*^ mice were crossed with *ROSA*^*mTmG/mTmG*^ mice, a Cre reporter strain in which Cre expressing cells would express GFP [49]. Resulting animals (*TyrCreER*^*T2*^ *PIKfyve*^*Flox/Flox*^ *ROSA*^*mTmG/+*^*)* or controls (*TyrCreER*^*T2*^ *ROSA*^*mTmG/+*^*)* were fed tamoxifen from p21 to p50, animals were sacrificed at p100 and frozen sections were examined by fluorescence microscopy. Both control and PIKfyve knockout mouse skin had GFP positive cells associated with hair follicles (Fig 3C) while the PIKfyve knockouts accumulated white hairs (Fig 3D), suggesting that PIKfyve deletion does not significantly affect the survival of melanocytes *in vivo*.

**Fig 4:**
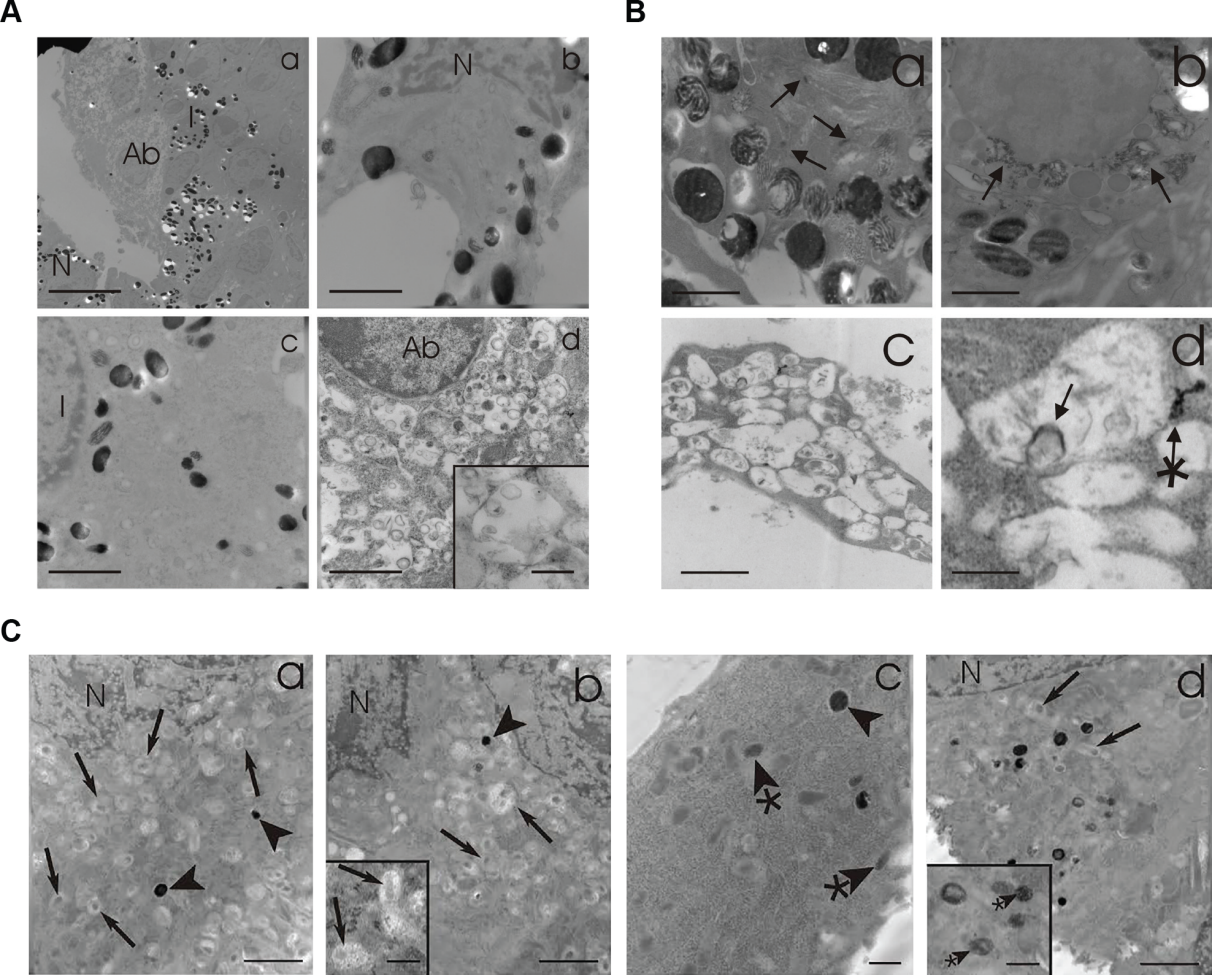
Melanocyte specific PIKfyve knockout mice exhibit abnormal melanocyte morphology and trafficking. **A**) 4mm biopsies were obtained from PIKfyve knockout mice and processed for electron microscopy. (a) Anagen hair bulbs contained melanocytes in various conditions, i.e. normal (N), abnormal (Ab), and intermediate (I). (b) Approximately 40% appeared morphologically normal resembling follicular melanocytes in C57Bl mice (N). (d) Another 40% exhibited profound vacuolization exhibiting vesicles within vacuoles resembling multivesicular bodies (inset to d) with very few if any melanosomes. (c) Occasionally, approximately 20% melanocytes relatively fewer melanosomes generally of earlier stages than the morphologically normal melanocytes. BARS: a = 10 microns, b, c & d = 3.0 microns, inset to d = 0.75 microns. **B)** 4mm biopsies were obtained from PIKfyve knockout mice and processed for DOPA histochemistry and electron microscopy. (a) morphologically normal melanocytes exhibited minimal DOPA reaction product in the trans Golgi network and associated 50 nm vesicles (arrows). (b) Intermediate melanocyte exhibited uncharacteristic DOPA reaction product that was clustered in large vacuoles some of which contained filamentous material (arrows). (c) The vacuoles of abnormal melanocytes exhibited very little DOPA reaction product with an occasional deposition around vesicles within the vacuoles (arrow) and attached to the limiting membrane of the vacuole (arrow with asterisk). BARS: a, b & c = 1.0 microns, d = 0.3 microns. **C)** Ultrastructure of melanocytes cultured from PIKfyve knockout mice recapitulate the aberrant morphology observed in hair bulb melanocytes from PIKfyve knockout mice. a & b) A prominent number of cultured melanocytes exhibited vacuoles with a central core of amorphorus material (arrows) with occasional stage IV melansomes (arrowheads). c & d) A significant number of melanocytes exhibited many stage IV melanosomes (arrowheads) and earlier stage melanosomes with normally arraigned melanofilaments (arrowheads with asterisks) as well as few vacuoles with a central core of amorphorus material (arrows). N = nucleus. Bars: a-d = 5 microns, insets = 2 microns.

Published studies have demonstrated that PIKfyve is essential for vesicular trafficking as loss or inhibition results in severe trafficking defects and vacuolization [21, 28, 33, 38]. Other studies have also demonstrated that PIKfyve is essential for lysosomal trafficking [24, 50, 51] and in lysosomal reformation from endolysosomes [36]. To further characterize PIKfyve’s role in melanosome biogenesis, skin biopsies were taken at p60 and assessed at the ultrastructural level by electron microscopy and DOPA histochemistry electron microscopy (EM). Intriguingly, melanocytes from knockout mice exhibit three morphological phenotypes when compared to controls (Fig 4A, subpanel a). Some of the melanocytes were phenotypically normal (N) (Fig 4A, subpanel b) while some exhibited profound vacuolization exhibiting vesicles within vacuoles resembling multivesicular endosomes (Fig 4A, subpanel d inset) plus very few melanosomes (Ab) (Fig 4A, subpanel d). Still others had an intermediate phenotype containing fewer melanosomes (I) (Fig 4A, subpanel c). Similarly, after DOPA incubation, morphologically normal cells contain minimal melanin reaction product in the Golgi area (Fig 4B, subpanel a), while uncharacteristic deposition was observed in intermediate cells (Fig 4B, subpanel b). The abnormal cells had less TYR reaction product (Fig 4B, subpanel c). Closer examination of the grossly abnormal melanocytes revealed the accumulation of single membrane vesicles that had multiple vesicles within them and some TYR reaction product, reminiscent of multivesicular endosomes (Fig 4B, subpanel d). To understand whether the variable phenotypes observed *in vivo* were a consequence of partial/incomplete loss of PIKfyve, we cultured melanocytes from *TyrCreER*^*T2*^ *PIKfyve*^*Flox/Flox*^ mice, treated them with tamoxifen or vehicle, and examined the structure of melanosomes in the cultured cells by electron microscopy. Electron microscopy analysis indicated that some tamoxifen treated *TyrCreER*^*T2*^ *PIKfyve*^*Flox/Flox*^ melanocytes accumulated single membrane structures resembling multivesicular endosomes with few stage IV melanosomes (Fig 4C, subanel a,b inset). Other treated melanocytes accumulated early and late stage melanosomes with a few single membrane structures (Fig 4C, subpanel c,d).

### Pharmacologic inhibition of PIKfyve blocks melanosome maturation

**Fig 5:**
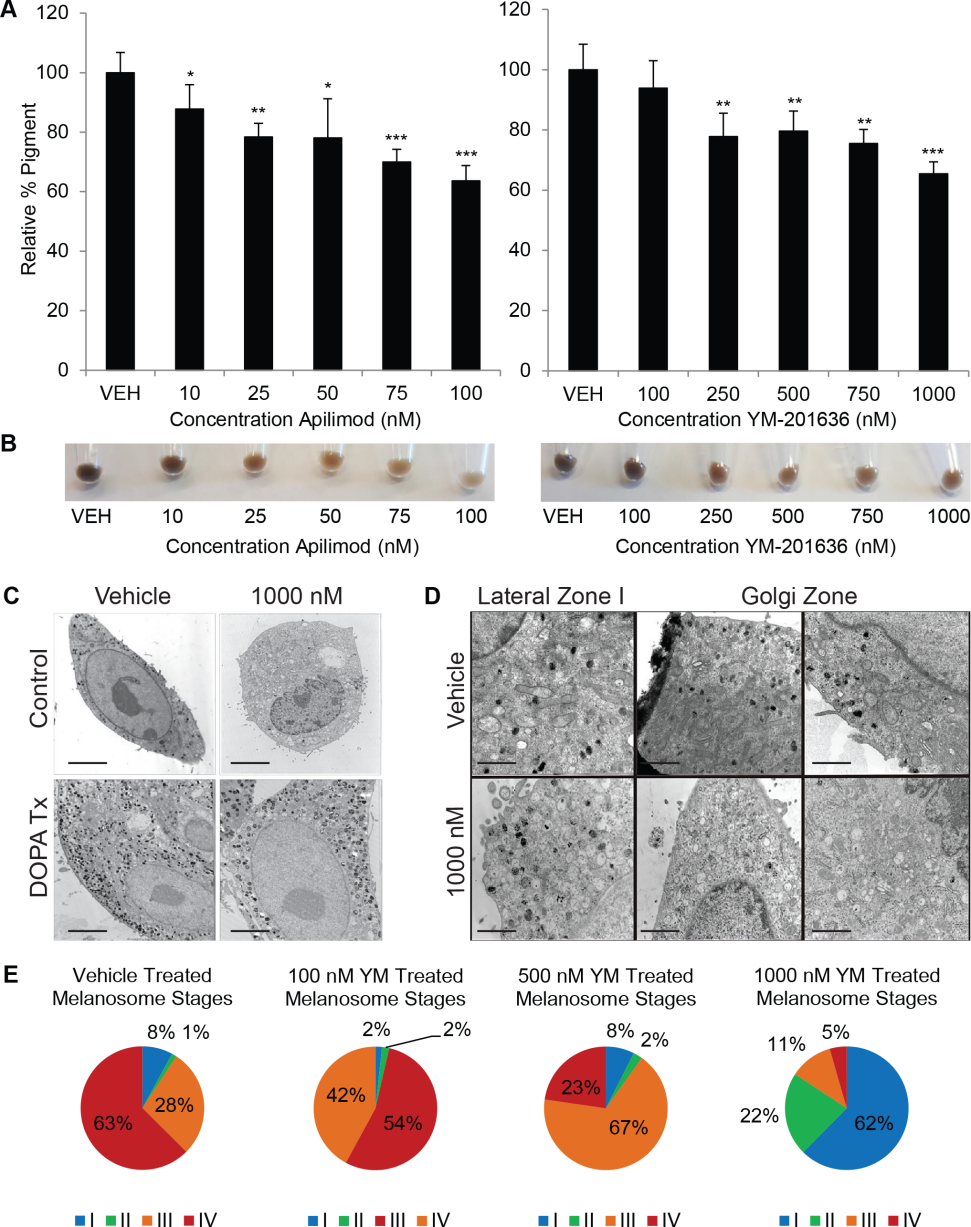
PIKfyve inhibition results in decreased melanin accumulation and decreased accumulation of advanced stage melanosomes. A) MNT-1 cells were treated with PIKfyve inhibitors YM-201636 (YM), apilimod or vehicle control for five days. A spectrophotometric melanin quantitation assay was used to measure the amount of accumulated melanin. (n = 6 as indicated by error bars). B) Equal numbers of MNT-1 cells treated with the indicated doses of YM or Apilimod were pelleted. Photographs of the cell pellets were obtained to document that YM and Apilimod treatment inhibited the accumulation of melanin. Normal human melanocytes (NHM) were treated with 1000 nM YM-201636 or vehicle without (Control) or with DOPA histochemistry (DOPA Tx) and (C,D) observed by electron microscopy Scale bar, C = 5 μm and D = 2 μm. (E) Pie graphs representing quantification of melanosome stages as percentage in NHM treated with 100, 500, or 1000 nM YM-021636 or vehicle. Each experiment was performed three times in triplicate. For all experiments, data shown are mean ± S.D *, p < 0.05; **, p < 0.01; or ***, p < 0.001 using a Student’s paired T test versus vehicle treated control.

Initial studies verified that loss of PIKfyve inhibits the normal maturation of melanosomes *in vivo* (Figs 1–4). To more accurately determine how PIKfyve modulates melanosome maturation, we examined the consequences of pharmacologic inhibition of PIKfyve *in vitro*. Published studies have verified that pharmacologic inhibition of PIKfyve potently and acutely blocks enzymatic activity [38, 50] and significantly reduces phosphoinositide levels [38]. MNT-1 melanoma cells that produce higher amounts of melanin than normal melanocytes *in vitro* were treated with two PIKfyve inhibitors, YM-201636 (YM) and apilimod, the latter of which has been noted for increased potency and specificity [30]. MNT-1 cells treated with YM or apilimod accumulated less melanin as compared to vehicle treated cells (Fig 5A and 5B), similar to what was observed in experimental mice and cells from experimental mice (Figs 1 and 2).

Once we determined that pharmacologic inhibition of PIKfyve resulted in decreased melanin production, we sought to determine how inhibition of PIKfyve blocks melanosome biogenesis at the ultrastructural level. Established cultures of darkly pigmented human melanocytes (DP melanocytes) were treated with various dosages of YM and processed for routine and DOPA histochemistry electron microscopy. YM (1000 nM) treated melanocytes were dramatically hypopigmented in comparison to vehicle treated cells (Fig 5C). Upon DOPA incubation, melanosomes in the treated melanocytes contained melanin reaction product almost to the extent of control melanocytes (Fig 5C). However, the melanin deposition within melanosomes in treated melanocytes appeared irregular and less homogeneous. Higher magnification images of vehicle versus YM treated cells demonstrated that within the Golgi zone, melanosomes of all stages existed in the control, whereas primarily Stage I and a few Stage II melanosomes existed in the YM treated melanocytes (Fig 5D). In regions lateral to the Golgi zone and within dendrites, all stages of melanosomes existed in the YM treated melanocytes as opposed to predominantly Stage IV in the control treated melanocytes (Fig 5D). Quantification of melanosome stages treated with YM demonstrated that high doses lead to an increase in stage I melanosomes and a decrease in stage IV melanosomes (Figs 5E and S3A). Taken together, these results suggest that PIKfyve influences the maturation of stage I and II melanosomes.

Once we realized that the percentage of “stage I” melanosomes increased with YM treatment, these primitive organelles were subjected to further scrutiny. It has been demonstrated that tyrosinase exits the Golgi in 50 nm trafficking vesicles. En route to the stage II melanosome, tyrosinase enters the multivesicular endosome (MVE) and then is rapidly re-recruited by a complex containing AP-3 into vesicles that ultimately transports this cargo to Stage II melanosomes [18]. Without DOPA incubation it is difficult to ultrastructurally discern these MVEs from Stage I melanosomes particularly when melanofibrils are not apparent. After DOPA incubation, the MVEs appear with DOPA reaction peripherally around their limiting membranes due to the fact that tyrosinase is a transmembrane enzyme whose catalytic carboxy end protrudes into the lumen (S3B Fig). These MVEs can occasionally appear in the Golgi zone of control melanocytes however subjectively many more appear in the YM treated melanocytes and possibly in the dendrites (S3B Fig). Melanosome density within both the cell body and the dendrites of control and YM treated melanocytes with and without DOPA treatment was quantified. There was a statistically significant increase in melanosome density in the cell body of YM treated melanocytes versus control in both the non-DOPA and DOPA treated group (S1 and S2 Tables). In contrast, no difference in melanosome density was observed in the dendrites. The density of 50 nm vesicles containing tyrosinase cargo was quantitated in DOPA processed melanocytes. In the Golgi area, the density of DOPA positive 50 nm vesicles was significantly increased in the YM versus the vehicle treated melanocytes (S3C Fig), indicating the accumulation of stage I melanosomes. Taken together, these studies suggest that PIKfyve inhibitors block melanosome maturation and the trafficking of tyrosinase from the MVE to the melanosome.

**Fig 6:**
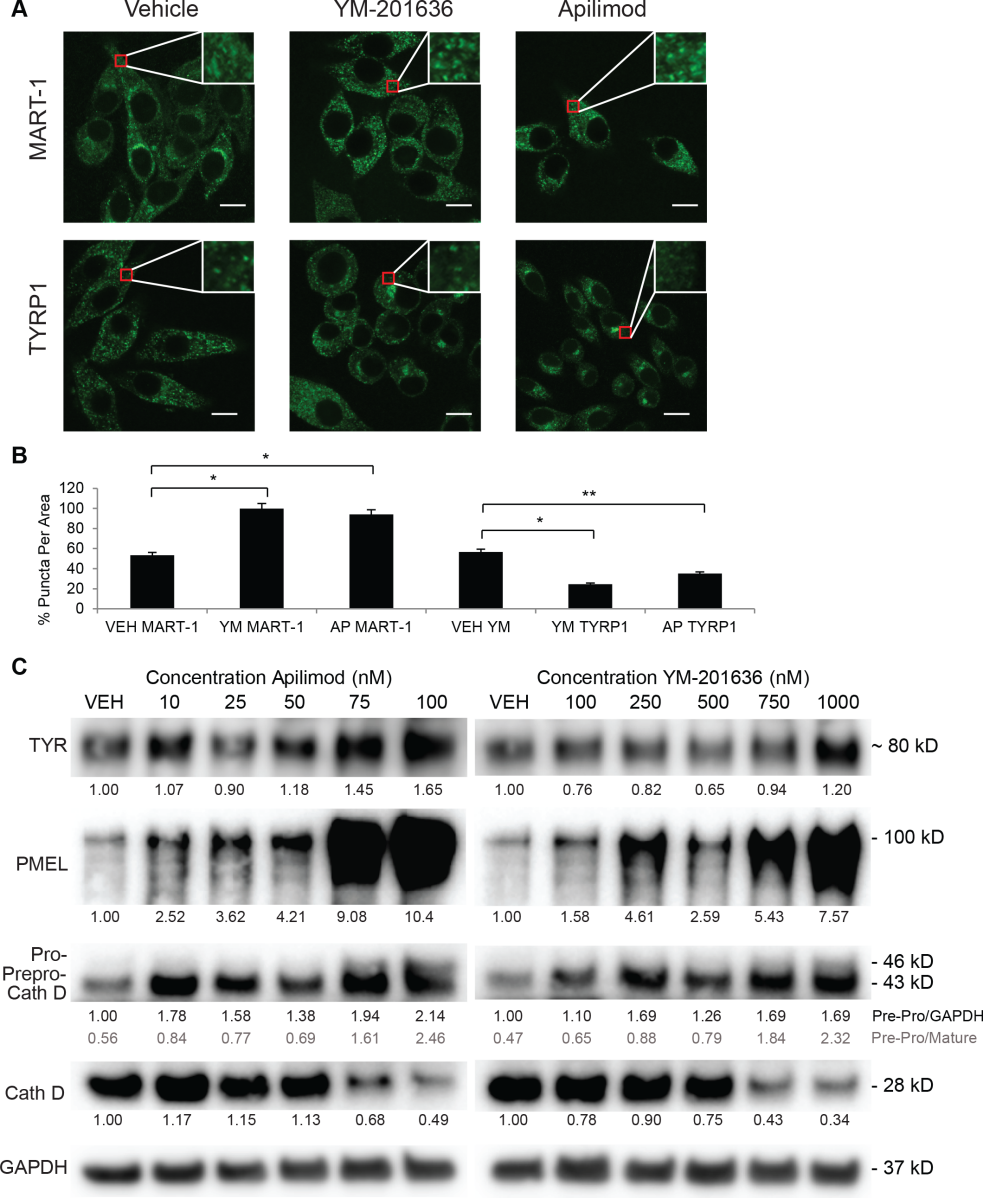
PIKfyve inhibition alters trafficking and processing of melanosomal proteins. A) MNT-1 cells were treated with 1000 nM YM-201636 (YM), 100 nM apilimod (AP), or vehicle (VEH) control for 16 hours. Cells were fixed and stained with anti- MART-1 or anti-TYRP1 antibodies and imaged by confocal microscopy depicted as GFP positive puncta. *Scale bar*, 10 μm. B) The number of GFP positive puncta in each MNT-1 cell was counted, and at least 20 cells were included for each group. The data are presented as the mean ± S.D. based on three independent experiments, data shown as percent signal relative to vehicle. For all experiments, data shown are mean ± S.D *, p < 0.05; **, p < 0.01; or ***, p < 0.001 using a Student’s paired T test versus vehicle treated control. C) MNT-1 cells were treated with PIKfyve inhibitors YM-201636, apilimod or vehicle control for 72 hours. The relative accumulation of TYR, and unprocessed PMEL (100 kD) and Cathepsin D was measured by immunoblotting. Protein accumulation relative to GAPDH levels was quantified by densitometry. Pre pro cathepsin D and pro cathepsin D was quantified relative to GAPDH level (black) as well as relative to mature Cathepsin D (gray). Representative experiment of three independent experiments is shown.

Next we sought to verify that PIKfyve regulates the maturation of the stage I to the stage IV melanosome by inhibiting the trafficking of proteins out of an intermediate endosomal compartment/MVE. To do this, we examined the accumulation of stage I and stage III/IV melanosome markers and the processing or accumulation of melanosome proteins. Previous studies demonstrated that stage I melanosomes containing MART-1 are primarily localized to the perinuclear region [52]. Immunofluorescence microscopy revealed that MART-1 positive vesicles accumulate in PIKfyve inhibitor treated MNT-1 cells (Fig 6A and 6B), consistent with the electron microscopy observation that stage I melanosomes accumulate in PIKfyve inhibitor treated primary melanocytes (Fig 5). Conversely, PIKfyve inhibitor treatment blocked the normal trafficking of TYRP1, resulting in the accumulation of TYRP1 containing vesicles in the perinuclear region and a lack of TYRP1 staining at the cell periphery (Fig 6A and 6B). Published studies have revealed that mature Tyrp1 containing melanosomes are localized to the cell periphery, while disruption of melanosome maturation results in the accumulation of Tyrp1 in an early endosome, which is located adjacent to the nucleus [16, 53]. These imaging results suggest that after PIKfyve inhibition, Tyrp1 is trapped in an early endosomal compartment (perinuclear localization), unable to reach the mature melanosome.

PMEL is cleaved during the formation of the stage II melanosome [7], resulting in the formation of PMEL fibrils that serve as a platform for melanin polymerization [12]. In order to determine whether PIKfyve affects the formation of the stage II melanosome, we determined whether PIKfyve inhibition or depletion inhibited the accumulation of full length PMEL. Increasing concentrations of PIKfyve inhibitor blocked the cleavage of PMEL protein resulting in the accumulation of full length PMEL protein while having little effect on tyrosinase accumulation (Fig 6C). Similarly, we observed that PIKfyve deletion in primary mouse melanocytes also resulted in the accumulation of full length PMEL protein (Fig 1B). In addition, PIKfyve inhibition blocked the accumulation of mature cathepsin D (Fig 6C), consistent with previously published studies indicating that PIKfyve inhibition blocks lysosomal processing of cathepsin [24] and lyososomal reformation [36].

PIKfyve inhibition is known to induce endosomal vacuolation within hours of treatment [54]. In this study, we sought to measure the effects of PIKfyve inhibition on melanosomes, vesicles that are generated from endosomes [3] that turn over slowly within the cell. To get a better understanding of the kinetics of the effect of PIKfyve inhibition on melanosome biogenesis, we measured the effects of PIKfyve inhibition on PMEL cleavage after treatment for 30 minutes, 1 hour, 2 hours, or 4 hours (S4A Fig). These studies indicated that the accumulation of full length PMEL could be observed 4 hours after treatment. Recently published studies indicated that PIKfyve inhibition can disrupt lysosome reformation, a phenotype that could be observed 6 hours after inhibitor treatment [36]. The observation that PIKfyve inhibition could induce changes in PMEL accumulation 4 hours after treatment is on par with the time scale required for lysosome disruption [36].

**Fig 7:**
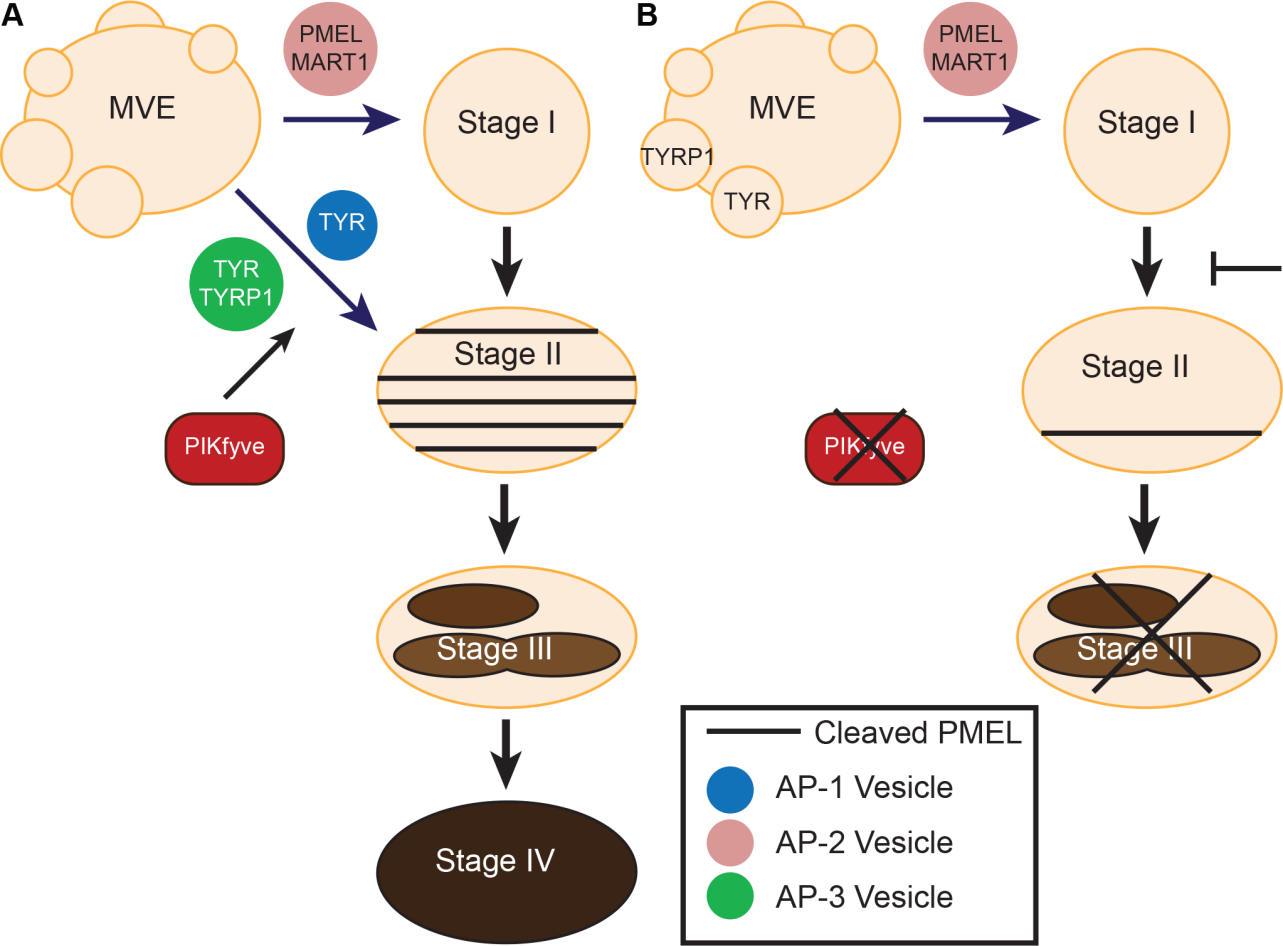
PIKfyve regulates melanosome maturation. A) In normal melanocytes, PIKfyve regulates melanosome biogenesis by controlling the delivery of TYR and TYRP1 to the melanosome and inducing PMEL processing. B) Inhibition or loss of PIKfyve blocks PMEL processing and prevents TYR and TYRP1 from being trafficked to stage II melanosomes.

Previously published studies demonstrate that PIKfyve inhibitor treatment induced cell death [55]. To verify that the PIKfyve inhibitors used here had similar cytotoxic effects, we examined the effect of PIKfyve inhibitors on MNT-1 cell viability. PIKfyve inhibitor treatment induced cell death in MNT-1 cells, an effect that could be rescued by the addition of PI(3,5)P_2_ but not PI(5)P or PI(3)P (S4B Fig). Taken together, these studies demonstrate that PIKfyve regulates the processing of PMEL and transport TYRP1 from an intermediate vesicle to the melanosome. In the absence of functional PIKfyve, PMEL, TYRP1, and TYR accumulate in a vesicular structure that resembles a MVE and are unable to get to the melanosome (Fig 7).

## Discussion

Published studies revealed that *VAC14 ingls* and *FIG4* pale tremor mice had lightening of the coat and severe neurologic disease, resulting in early lethality and the accumulation of autophagosomes within the CNS [35]. These phenotypes made it difficult to determine how exactly these mutations resulted in the coat color defects observed. Similarly, the *PIKfyve* gene-trap mouse also had severe neurologic disease, which ultimately resulted in early lethality [43], making it difficult to assess whether PIKfyve regulates melanogenesis. To circumvent these limitations and study the effects on adult melanocytes, we generated a melanocyte lineage specific PIKfyve-knockout mouse model that utilized a tyrosinase driven Cre recombinase [45]. Melanocytes were cultured from *TyrCreER*^*T2*^ *PIKfyve*^*Flox/Flox*^ mice and incubated in the presence and absence of tamoxifen (Fig 1). Tamoxifen treated melanocytes accumulated less melanosomes and less PIKfyve protein as compared to untreated cells (Fig 1B). While these cells accumulated less PIKfyve, PIKfyve was not completely absent as was observed in experiments where *PIKfyve*^*Flox/Flox*^ MEFs were infected with adenovirus expressing Cre recombinase [44].

Melanocyte-specific PIKfyve knockout mice accumulated grey hairs that were initially more obvious in shave depilated areas but that eventually were observed in hairs that were not shave depilated (Fig 2). This phenotype did not progress as the mice aged in contrast to other models of stem cell depletion [48]. *TyrCreER*^*T2*^ *PIKfyve*^*Flox/Flox*^ mice were crossed with a Cre reporter strain (*ROSA*^*mTmG*^) and treated with tamoxifen to determine whether PIKfyve deletion affected melanocyte viability *in vivo* (Fig 3C). Both PIKfyve knockout skin and control skin had GFP expressing melanocytes (Fig 3C), indicating that the observed phenotypes were not secondary to complete melanocyte loss. High magnification light microscopy images of mouse hair follicles revealed that not all of the hair follicles from PIKfyve knockout mice contained vacuolated cells (Fig 4C). Similarly, EM studies revealed that not all melanocytes from the animal accumulated abnormal endosomal vesicles (Fig 4A and 4B). Taken together, these results indicate that the mouse phenotype observed was not the result of complete deletion of PIKfyve but instead a partial loss of function phenotype. This allowed us to study the effects of PIKfyve depletion on melanosome biogenesis as melanocytes remained in the PIKfyve knockout animal.

As PIKfyve deficient melanocytes were still detectable in the adult animal (Fig 3C), we were able to then examine the consequences of PIKfyve depletion on melanosome biogenesis *in vivo*. Epidermal melanocytes from tamoxifen fed *TyrCreER*^*T2*^ *PIKfyve*^*Flox/Flox*^ mice accumulated abnormal single membrane vesicles with smaller vesicles within them reminiscent of MVEs that also contained tyrosinase reaction product (Fig 4A and 4B). A similar phenotype was observed when melanocytes from *TyrCreER*^*T2*^ *PIKfyve*^*Flox/Flox*^ were cultured and treated with tamoxifen (Fig 4C), and MVEs containing DOPA reaction product were also shown to accumulate in PIKfyve inhibitor treated primary melanocytes (S3 Fig). Similarly, some of the MVE-like structures in the animal had DOPA reaction product within them, suggesting that they contain TYR, which may be trapped within this compartment (Fig 4B). Consistent with these observations, immunofluorescence studies in MNT-1 cells demonstrated that PIKfyve inhibition blocked PMEL processing and the trafficking of TYRP1 to the melanosome (Fig 6). Other studies have shown that PI(3,5)P_2_ plays an essential role in MVE protein sorting and retrograde trafficking [31, 56, 57]. Specifically, PI(3,5)P_2_ has been shown to regulate ESCRT-III function and MVE trafficking [57, 58] downstream of ESCRT-I [59], which has been shown to be required for TYRP1 transport [16]. Interestingly, PIKfyve inhibition also affected the trafficking of MART-1 (Fig 6), whose proper trafficking requires ESCRT-I. Taken together, these results are consistent with a model where PIKfyve regulates the delivery of TYRP1 and TYR from the endosome/MVE to the terminal melanosome (Fig 7).

Recently published studies revealed that PI(3,5)P_2_ depletion inhibits the process of lysosome reformation from endolysosomes [36]. While we observed that PIKfyve inhibition affected lysosomal enzyme processing (Fig 6C), we observed a distinct phenotype in melanocytes- MVE accumulation. These results indicate that PI(3,5)P_2_ or PI(5)P that is generated from PI(3,5)P_2_ modulates the biogenesis of melanosomes in a way that is distinct from its effect on lysosomes. PI(3,5)P_2_ or PI(5)P could control the biogenesis of melanosomes in several different ways. These lipids could control the budding of vesicle cargo from the MVE en route to the melanosome or the fusion of vesicle cargo with the developing melanosomes. Alternatively, PI(3,5)P_2_ could be specifically required for ESCRT-I and ESCRT-III based trafficking of melanosome proteins. Finally, PI(3,5)P_2_ may regulate melanosome biogenesis by influencing conductance regulators required for pigmentation. Two families of cation channels, the TPCs and the TRPMLs, act as PI(3,5)P_2_ effectors and function in vesicular fusion [26, 60, 61]. In particular, TPC2 was found to be activated by PI(3,5)P_2_ and regulate pigmentation *in vitro* in an expression dependent context [25, 62, 63]. Furthermore, single-nucleotide polymorphisms in TPC2 have also been identified in humans that are correlated with skin, eye, and hair pigment variation [64]. Finally, mice mutant for TRPML3, another putative PI(3,5)P_2_ regulated channel, exhibit hypopigmentary phenotypes [65]. Future studies will define the specific phosphoinositide that regulates melanogenesis, determine how and when these phosphoinositides regulate melanosome maturation, and identify phosphoinositide effectors protein present on the melanosome that participate in this process.

## Materials and methods

### Ethics statement

All experiments involving mice conform to the NIH guidelines and were approved by the Institutional Animal Care and Use Committee (IACUC) of the University of California, Irvine, approval number 2011–3020.

### Antibodies and primers

All antibodies used in experimental assays are listed in S3 Table. *PIKfyve* genotyping primers and PCR parameters are described in [44]. Other genotyping primers and PCR settings were taken from the mouse mutant resource website, (Jackson Laboratory)).

### Cell culture

Human MNT-1 cells were cultured in DMEM (Genesee Scientific) supplemented with 15% fetal bovine serum (Corning), AIM-V medium (Life Technologies), MEM vitamin solution (Invitrogen), and antibiotic-antimycotic (Life Technologies). For melanin quantification experiments, MNT-1 cells were switched to DMEM lacking phenol red (Fisher Scientific) supplemented with 10% fetal bovine serum, L-glutamine (Invitrogen), and antibiotic-antimycotic. Human darkly pigmented neonatal epidermal melanocytes (Life Technologies) were cultured in Medium 254 (Life Technologies) supplemented with Human Melanocyte Growth Supplement 2 (Life Technologies) and antibiotic-antimycotic. Harvested primary melanocytes were grown in RPMI-1640 (Thermo Fisher Scientific) supplemented with 10% FBS, antibiotic-antimycotic, 200nM 12-O-tetradecanoylphorbol 13-acetate (TPA) (Abcam), and 200pM cholera toxin (Sigma-Aldrich) or RPMI-1640 supplemented with 5% FBS, 50ng/ml Stem cell Factor (SCF) (Protech International), 20nM Endothelin-3 (END3) (Sigma-Aldrich), 2.5ng/ml Fibroblast growth Factor (FGF) (Sigma-Aldrich), 100nM α-Melanocyte stimulating hormone (α-MSH) (Sigma-Aldrich), 1μM Phosphoethanolamine (Sigma-Aldrich), 10μM Ethanolamine (Sigma-Aldrich), and 1mg/ml Insulin (Sigma-Aldrich).

### Drug treatment

MNT-1 cells were plated in 6-well plates at a concentration of 2 x 10^5^ cells per well and allowed to adhere overnight. Cells were then incubated with varying concentrations of YM-201636 (Cayman Chemical), Apilimod (US Biological), or vehicle control (DMSO) in normal MNT-1 media. At the conclusion of the treatment, cells were lysed with RIPA buffer for downstream analysis. In treatment assays that exceeded 48 hours, media containing drug was refreshed every 48 hours.

### Pigment measurement

MNT-1 cells were plated in 96-well plates at a concentration of 1.5 x 10^4^ cells per well and allowed to re-attach overnight. Media was refreshed for drug treated cells every 48 hours. After five days of treatment cells were lysed with Cell-Titer-Glo reagent (Promega). Relative melanin accumulation was quantified by measuring absorbance at 405 nm and normalizing this value to luminescence to determine cell number as determined by the Cell-Titer-Glo assay as previously described [66]. Pigment percent was quantified relative to vehicle control. A Student’s two-tailed t-test was used to calculate the statistical significance in comparison to vehicle-treated control.

### PI Lipid treatment

All carriers and phospholipids were obtained from Echelon Biosciences. Unlabeled PI(5)P, PI(3)P and PI(3,5)P_2_ were reconstituted in DMSO:H_2_O (10:1); carrier 2 and carrier 3 were reconstituted in H_2_O. Carriers and lipids were combined at 1:1 molar ratio and incubated for 15 minutes at room temperature. Charge matched carriers were used to optimize lipid delivery–carrier 3 was used as a control for PI(5)P; carrier 2 was used as a control PI(3,5)P_2_. The mixture was then diluted in MNT-1 media and incubated with cells for 48 hours. The relative survival of cells in the presence and absence of lipid or carrier and PIKfyve inhibitor was measured using a Cell Titer-Glo assay. MNT-1 cells were plated in 96-well plates at a concentration of 1.5 x 10^4^ cells per well and allowed to re-attach overnight. Media containing drug and lipid was refreshed on cells every 48 hours. After five days of treatment cells were lysed with Cell-Titer-Glo reagent (Promega). The luminescence value was used to determine cell survival as determined by the Cell-Titer-Glo assay as previously described [67]. A Student’s two-tailed t-test was used to calculate the statistical significance in comparison to vehicle-treated control.

### Electron microscopy

Cultured melanocytes or whole mouse skin (*n* = 2 per genotype) harvested from either anesthetized or euthanized mice using a 4-mm round punch biopsy were fixed in half-strength Karnovsky’s fixative (Karnovsky, 1965) for 24 hours before being transferred to sodium cacodylate buffer, 0.2M, pH 7.4 (Electron Microscopy Sciences). Tissue was then postfixed with 1% osmium tetroxide containing 1.5% potassium ferrocyanide. After being dehydrated, tissues were embedded in EPON and sections were obtained using a RMC-MT6000XL ultramicrotome and stained with uranyl acetate and lead citrate. Sections were viewed and selected images were digitally photographed using a JEOL JEM-1230 transmission electron microscope. For DOPA histochemistry and prior to postfixation, cells or tissues were incubated in a 0.1%solution of l-DOPA twice for 2.5 hours. The cells and tissues were washed and processed as described above.

Darkly pigmented melanocytes were treated with various dosages of YM-201636 (0–1000 nM) for 72 hours. Cells were then fixed for 4 hours in Karnovsky’s fixative, pH 7.2, before being washed with sodium cacododylate buffer (0.2 M). Primary melanocytes were fixed for 30 minutes in Karnosky’s fixative, pH 7.2, before being washed with sodium cacododylate buffer (0.2M). Samples were then processed for routine DOPA histochemistry electron microscopy. Melanosome stages (I–IV) were quantified visually in the electron micrographs and melanosome stage percentage was assessed versus vehicle treated controls. Electron microscopy on whole mouse skin was obtained and processed as previously described by (48).

### Immunofluorescence microscopy

MNT-1 cells were plated in 12-well plates on coverslips at a concentration of 1 x 10^4^ cells per well and allowed to adhere overnight. Cells were then treated with 100 nM Apilimod, 1000 nM YM-201636, or vehicle control overnight. Alternatively, cells were treated with charge matched carrier or PI(3,5)P_2_ for 8 hours. Cells were fixed with 4% paraformaldehyde for 1 hour. Coverslips were rinsed with PBS and permeablized with 0.1% Triton X-100 (Fisher Scientific) and subsequently blocked in 2% BSA in PBS containing 0.1% Tween 20 for 1 hour. Cells were then incubated with primary antibodies (S3 Table) followed by secondary antibodies conjugated to Alexa Fluor 594 (Invitrogen) and were mounted in a solution containing DAPI. Confocal images were acquired using a LSM 780 confocal multiphoton microscope and images were processed in Zen lite (Zeiss). For imaging mouse skin from *TyrCreER*^*T2*^ *PIKfyve*^*Flox/Flox*^ *ROSA*^*mTmG/+*^, mice was harvested, skin was frozen in OCT blocks and cryosectioned. 4μm sections were imaged using a Nikon Eclispse Ti fluorescent microscope.

### Mouse strains and genotyping

All experiments involving mice conform to the NIH guidelines and were approved by the Institutional Animal Care and Use Committee (IACUC) of the University of California, Irvine, approval number 2011–3020. C57BL/6 *PIKfyve*^*Flox/Flox*^ mice on a pure C57BL/6 background were obtained from Dr. Takehiko Sasaki (Akita University, Akita, Japan). *PIKfyve*^*Flox/Flox*^ were crossed to *Tyrosinase*::*CreER*^*T2*^ (JAX stock no: 012328) on a pure C57BL/6 background. The resulting *Tyrosinase*::*CreER*^*T2*,^ *PIKfyve*^*Flox/+*^ progeny were backcrossed to *PIKfyve*^*Flox/Flox*^ to generate *Tyrosinase*::*CreER*^*T2*,^ *PIKfyve*^*Flox/Flox*^ mice. Upon weaning, mice were placed on tamoxifen feed (Harlan Laboraties, 250 mg/kg) for 29 days. Genomic DNA was isolated from mouse tail biopsies using the Quick Genotyping DNA Preparation Kit (Bioland Scientific, LLC) according to the manufacturer’s instructions. *Tyrosinase*::*CreER*^*T2*,^ *PIKfyve*^*Flox/Flox*^ mice were crossed with *ROSA*^*mTmG/mTmG*^ mice obtained from Jackson laboratories. Genotypes of progeny was determined using specific genotyping primers (S4 Table) using guidelines provided by Jackson laboratories. Resulting *TyrCreER*^*T2*^ *PIKfyve*^*Flox/Flox*^ *ROSA*^*mTmG/+*^ progeny were similarly placed on tamoxifen feed for 29 days as has been described above.

### Primary melanocyte isolation

Primary mouse melanocytes were collected based on methods described by Godwin et al. [68]. In brief, newborn mice less than 3 days old were sacrificed and sterilized. The skin was removed and cleaned of muscle. To dissociate the epidermis, the skin was incubated for 1 hour in 5mg/ml trypsin (Sigma-Aldrich) at 37°C. The skin was washed and the epidermis was split off. The epidermis was chopped in 0.25% trypsin-EDTA solution (Gibco) and resuspended in growth media. The resuspended cells were filtered using an 100μm cell strainer (Falcon) and plated on 10 cm dishes. Once melanocytes were established, TPA concentration was increased to 400nM to treat fibroblast contamination and increase pigmentation. To increase purity of melanocytes for later experiments, cells isolated from epidermis were incubated on CD117 MicroBeads (Miltenyi Biotec) and sorted on MACS LS columns (Miltenyi Biotec). CD117+ melanocytes were then plated in 24-well dishes and incubated in media containing 5% FBS, SCF, END3, FGF, α-MSH, Phosphoethanolamine, ethanolamine, and insulin.

### Primary melanocyte treatment and microscopy

Primary melanocytes were plated on 4-chambered coverglass (Thermo Fisher Scientific) and treated with 1μg/ml 4-hydroxytamoxifen (4-HTA) for 48hrs. Cells were fixed in 2% PFA for 1 hour at room temperature and imaged. Phase contrast images were acquired using a Nikon Eclipse Ti fluorescent microscope.

### Mouse hair

Dorsal hairs of mice at P50, P100, or P365 were shaved and 1 mg was dissolved overnight in 1 mL of 9:1 Soluene-350 (PerkinElmer) and water. Quadruplicate 150 μL aliquots for each mouse hair sample were then analyzed for absorbance values at 405 nm as previously described [47].

## Supporting information

S1 Fig Melanocyte specific PIKfyve knockout mice exhibit hair greying. (A) Representative photographs of female littermates of p35 *Tyrosinase*::*Cre*^*ERT2*^; *PIKFYVE*^*Flox/Flox*^ littermates. Cre- and Cre+ mice were administered tamoxifen (or normal) feed from day 21 to 35. (B) Representative photographs of individual littermates from each group taken at P85 or P105. (C) Side-by-side comparison of representative female littermates photographed at P105 or P365. (D) Mice were administered normal or tamoxifen containing feed for 29 days (P21-P50) after which all mice were administered normal feed. Mice were weighed at P365 and average weight of mice was calculated. For all experiments, all data are mean ± S.D. (n = 3 as indicated by error bars).
(TIF)

S2 Fig PIKfyve inhibition does not alter hair cycle of mice. (A) *Tyrosinase*::*Cre*^*ERT2*^; *PIKFYVE*^*Flox/Flox*^ littermates were administered normal or tamoxifen (TAM) containing feed for 21 days (P28-P50). At P50, mice were photographed, and then shaved and depilated at to stimulate the 3^rd^ hair cycle. By P60 mice were again photographed showing the regrowth of white hairs. (B) H&E staining of skin collected from mice at P60 imaged at 4x and 10x magnification. All visible hairs from mice fed a normal diet are pigmented. In contrast, mice fed TAM have both pigmented and unpigmented hairs in the skin. Black arrows denote unpigmented hairs.
(TIF)

S3 Fig PIKfyve inhibition results in decreased number of advanced stage melanosomes. (A) Quantification of melanosome stages as percentage in NHM treated with 100, 500, or 1000 nM YM-021636 or vehicle. (B) Darkly pigmented (DP) melanocytes were treated with 1000 nM YM-201636 or vehicle and observed by electron microscopy. Arrows indicate multivesicular endosomes that after DOPA histochemistry appear to have reaction product peripherally around their limiting membranes. *Scale bar*, 2 μm. (C) DP melanocytes were treated with 1000 nM YM-201636 or vehicle, processed for DOPA histochemistry and observed by electron microscopy. The density of DOPA positive 50nm vesicles in the Golgi area was quantitated. *Scale bar*, 2 μm. Numeration of melanosome density if the cell body and dendrite of NHM treated with 1000 nM YM-201636 or vehicle without or with DOPA histochemistry and P values determined by Student t-Test of the density data.
(TIF)

S4 Fig A) MNT-1 cells were treated with PIKfyve inhibitors YM-201636, apilimod or vehicle control for 30, 60, 120, or 240 minutes as indicated. The protein level of unprocessed PMEL (100 kDa) was accessed via immunoblotting and quantified by densitometry analysis relative to β-Actin. Each experiment was performed with three biological replicates and three technical replicates. B) MNT-1 cells were treated with 1000 nM YM-201636 or vehicle control for five days and 0.5 μM of phospholipids [PI(3)P, PI(3,5)P2, PI(5)P] or carrier alone. Relative cell survival was quantified using a Cell-Titer-Glo assay.
(TIF)

S1 Table Numeration of melanosome density of normal human melanocytes treated with YM-201636 or vehicle. Melanocytes were treated with vehicle or 1000nM YM-201636 and analyzed with or without DOPA histochemistry. The average number of melanosomes per 100μm^2^ were calculated for both the cell body and dendrites of the melanocytes.
(DOCX)

S2 Table P-values for pairwise comparisons of the average number of melanosomes in the cell body or dendrite of melanocytes presented in S1 Table. (DOCX)

S3 Table List of antibodies and dilutions used in experiments. WB = Western Blot. IF = Immunofluorescence.
(DOCX)

S4 Table List of primers used to genotype mice for experiments and their sequences, 5’ to 3’. For = Forward primer. Rev = Reverse primer.
(DOCX)
