# Supplementary figures and images for: PIKfyve regulates melanosome biogenesis
Source: PLoS Genet. 2018 Mar 27;14(3):e1007290. doi: 10.1371/journal.pgen.1007290 (PMC5889185; doi:10.1371/journal.pgen.1007290)

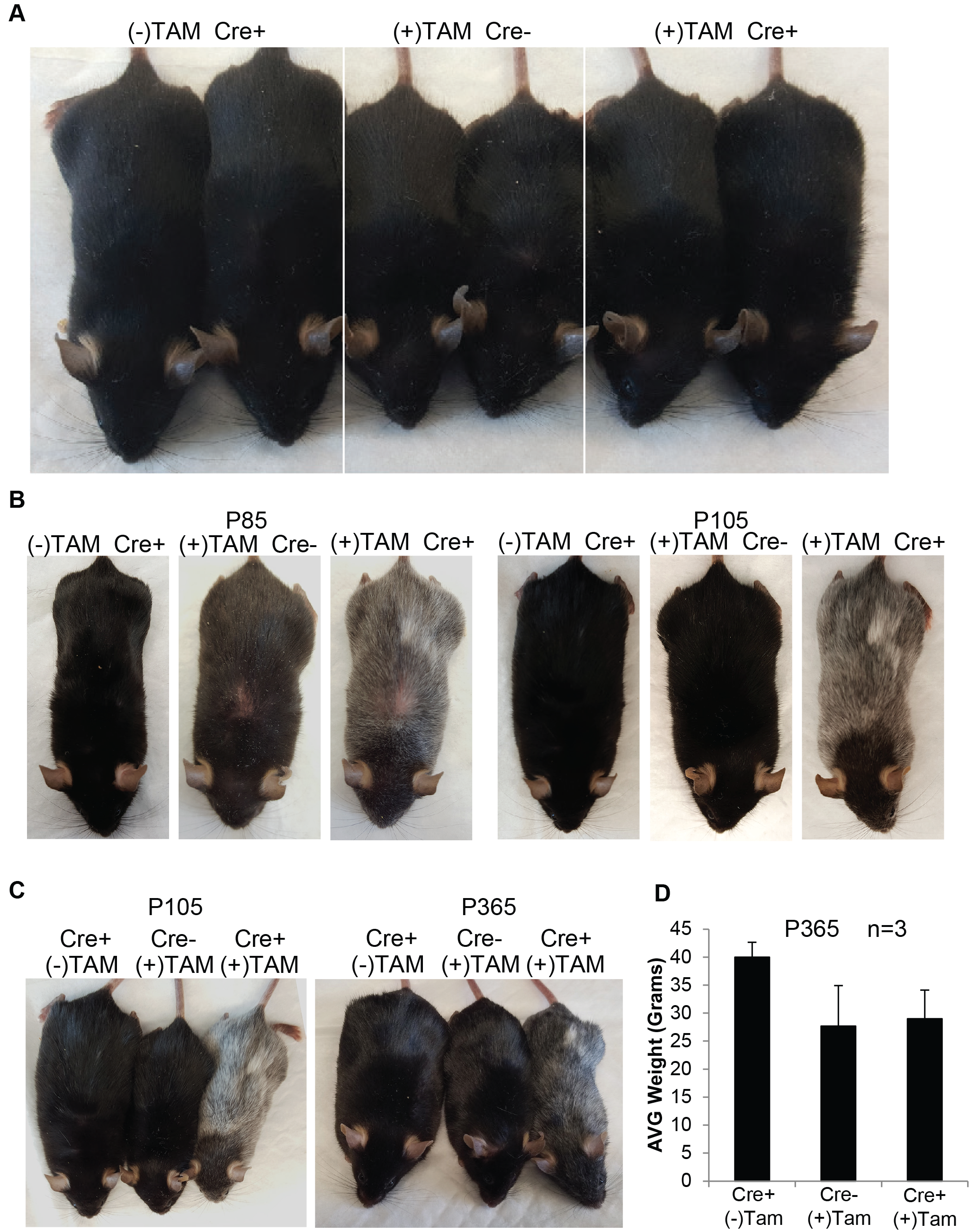

Supplement: S1 Fig — (A) Representative photographs of female littermates of p35 Tyrosinase::CreERT2; PIKFYVEFlox/Flox littermates. Cre- and Cre+ mice were administered tamoxifen (or normal) feed from day 21 to 35. (B) Representative photographs of individual littermates from each group taken at P85 or P105. (C) Side-by-side comparison of representative female littermates photographed at P105 or P365. (D) Mice were administered normal or tamoxifen containing feed for 29 days (P21-P50) after which all mice were administered normal feed. Mice were weighed at P365 and average weight of mice was calculated. For all experiments, all data are mean ± S.D. (n = 3 as indicated by error bars). (TIF) [file pgen.1007290.s001.tif]

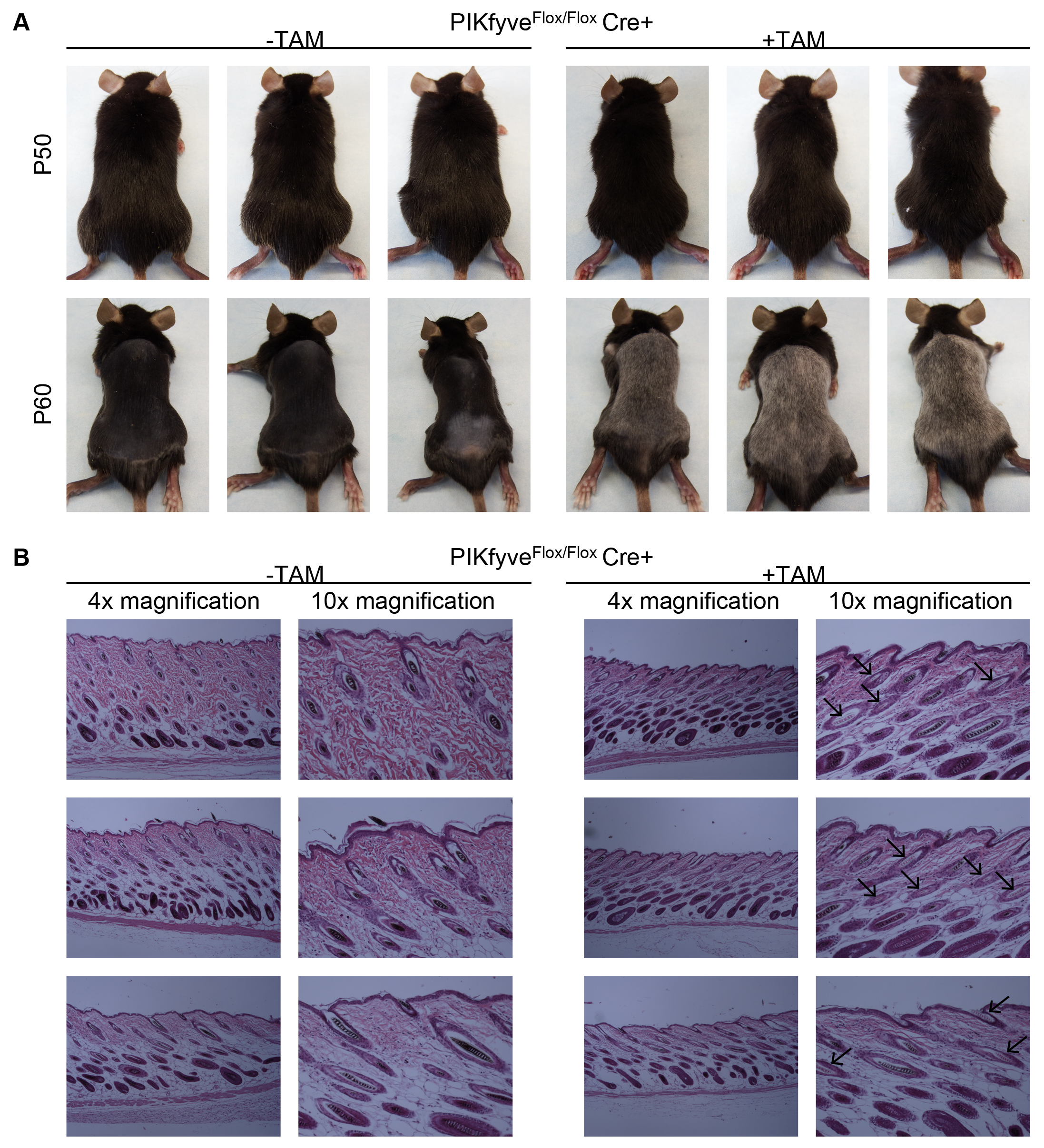

Supplement: S2 Fig — (A) Tyrosinase::CreERT2; PIKFYVEFlox/Flox littermates were administered normal or tamoxifen (TAM) containing feed for 21 days (P28-P50). At P50, mice were photographed, and then shaved and depilated at to stimulate the 3rd hair cycle. By P60 mice were again photographed showing the regrowth of white hairs. (B) H&E staining of skin collected from mice at P60 imaged at 4x and 10x magnification. All visible hairs from mice fed a normal diet are pigmented. In contrast, mice fed TAM have both pigmented and unpigmented hairs in the skin. Black arrows denote unpigmented hairs. (TIF) [file pgen.1007290.s002.tif]

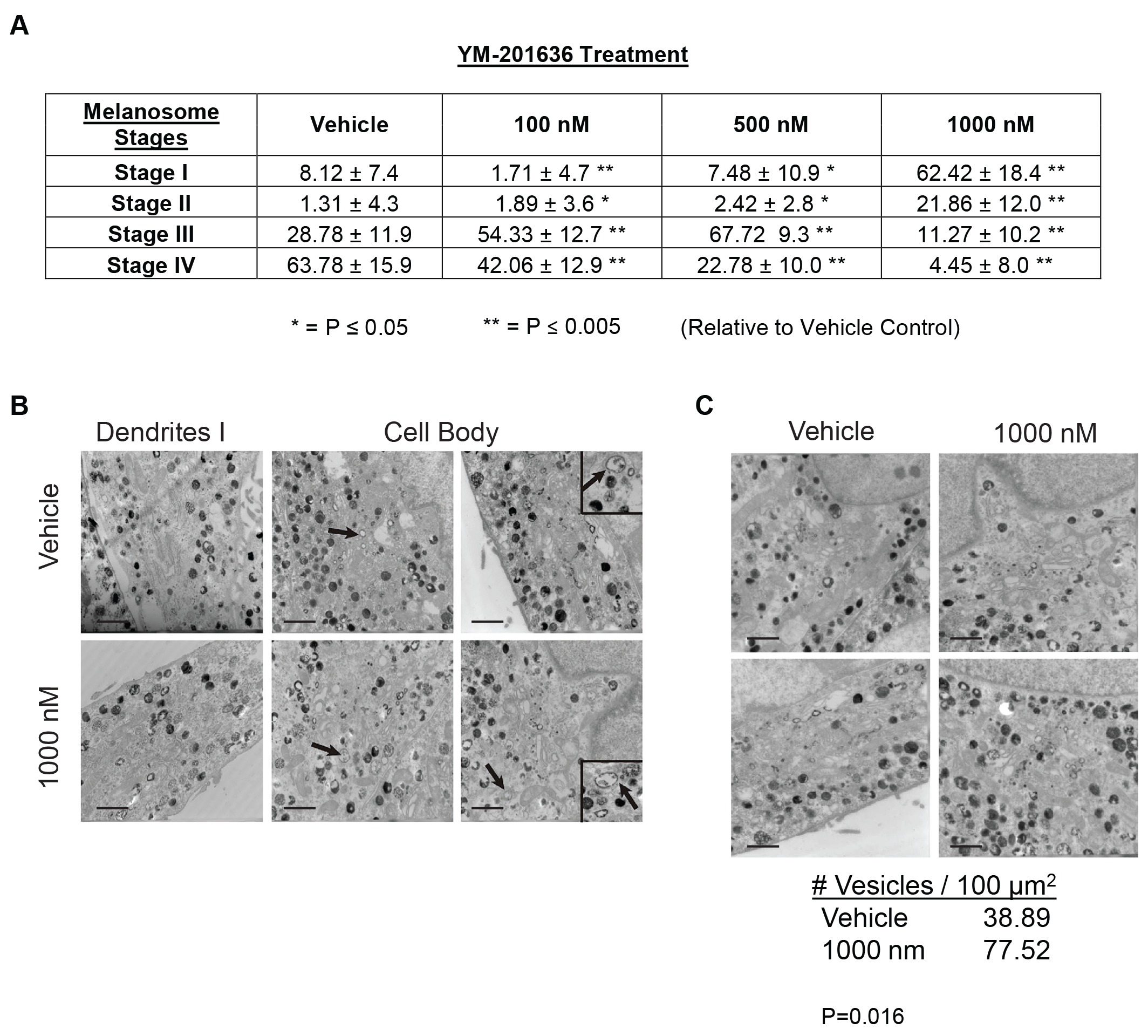

Supplement: S3 Fig — (A) Quantification of melanosome stages as percentage in NHM treated with 100, 500, or 1000 nM YM-021636 or vehicle. (B) Darkly pigmented (DP) melanocytes were treated with 1000 nM YM-201636 or vehicle and observed by electron microscopy. Arrows indicate multivesicular endosomes that after DOPA histochemistry appear to have reaction product peripherally around their limiting membranes. Scale bar, 2 μm. (C) DP melanocytes were treated with 1000 nM YM-201636 or vehicle, processed for DOPA histochemistry and observed by electron microscopy. The density of DOPA positive 50nm vesicles in the Golgi area was quantitated. Scale bar, 2 μm. Numeration of melanosome density if the cell body and dendrite of NHM treated with 1000 nM YM-201636 or vehicle without or with DOPA histochemistry and P values determined by Student t-Test of the density data. (TIF) [file pgen.1007290.s003.tif]

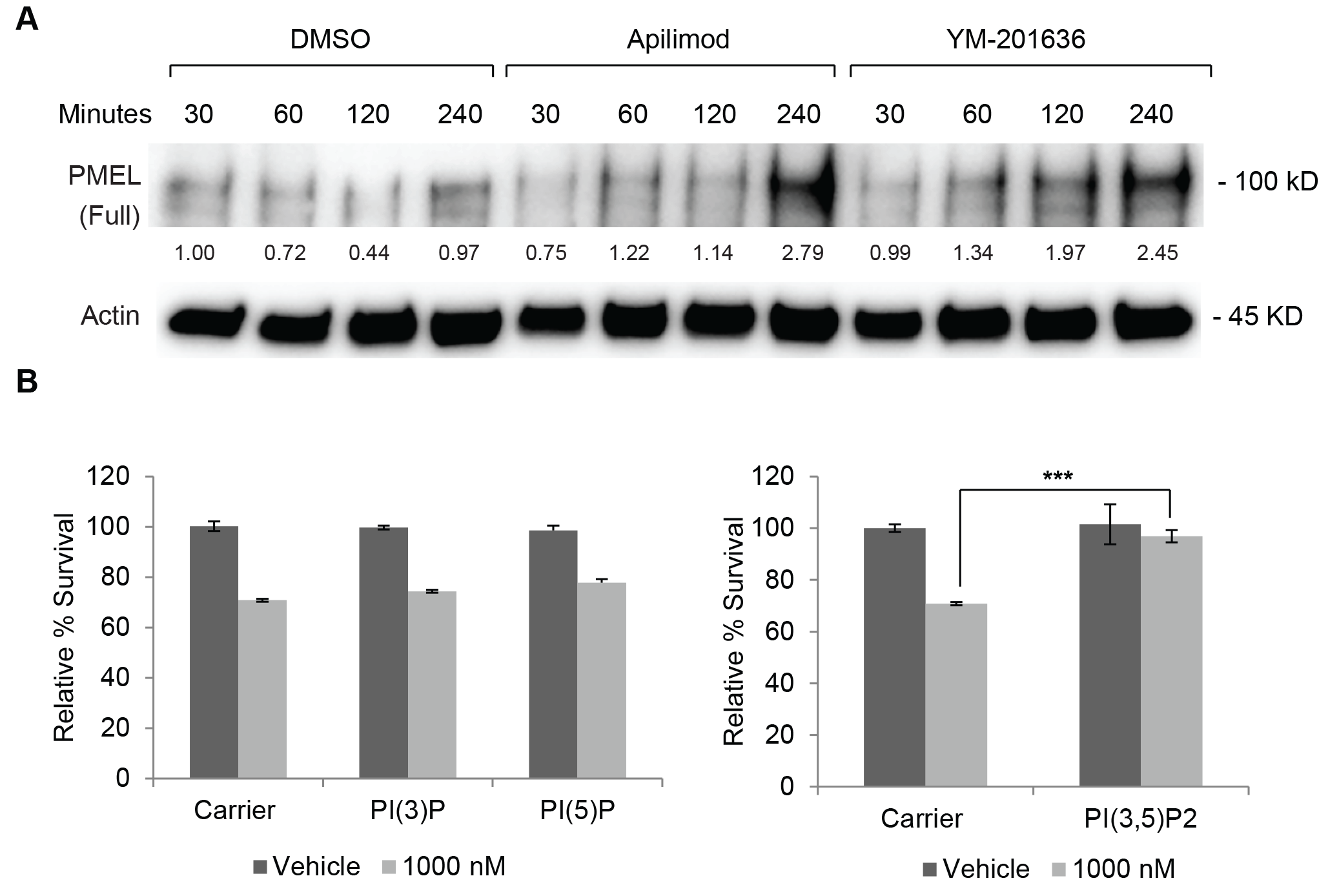

Supplement: S4 Fig — A) MNT-1 cells were treated with PIKfyve inhibitors YM-201636, apilimod or vehicle control for 30, 60, 120, or 240 minutes as indicated. The protein level of unprocessed PMEL (100 kDa) was accessed via immunoblotting and quantified by densitometry analysis relative to β-Actin. Each experiment was performed with three biological replicates and three technical replicates. B) MNT-1 cells were treated with 1000 nM YM-201636 or vehicle control for five days and 0.5 μM of phospholipids [PI(3)P, PI(3,5)P2, PI(5)P] or carrier alone. Relative cell survival was quantified using a Cell-Titer-Glo assay. (TIF) [file pgen.1007290.s004.tif]
