# Supplementary material for: PIKfyve regulates melanosome biogenesis
Source: PLoS Genet. 2018 Mar 27;14(3):e1007290. doi: 10.1371/journal.pgen.1007290 (PMC5889185; doi:10.1371/journal.pgen.1007290)
Supplement: S1 Table — Melanocytes were treated with vehicle or 1000nM YM-201636 and analyzed with or without DOPA histochemistry. The average number of melanosomes per 100μm2 were calculated for both the cell body and dendrites of the melanocytes. (DOCX) [file pgen.1007290.s005.docx]

**S1 Table**

| **Treatment** | **Histochemistry** | **Cell Area** | **Average # melanosomes/100 µm^2^** |
| --- | --- | --- | --- |
|  |  |  |  |
| Vehicle | No DOPA | Cell Body | 129 |
| Vehicle | DOPA | Cell Body | 139 |
| 1000 nM YM-201636 | No DOPA | Cell Body | 171.3 |
| 1000 nM YM-201636 | DOPA | Cell Body | 179.4 |
| Vehicle | NO DOPA | Dendrite | 142.5 |
| Vehicle | DOPA | Dendrite | 138.5 |
| 1000 nM YM-201636 | No DOPA | Dendrite | 139.5 |
| 1000 nM YM-201636 | DOPA | Dendrite | 140.6 |
