# Supplementary material for: PIKfyve regulates melanosome biogenesis
Source: PLoS Genet. 2018 Mar 27;14(3):e1007290. doi: 10.1371/journal.pgen.1007290 (PMC5889185; doi:10.1371/journal.pgen.1007290)
Supplement: S2 Table — (DOCX) [file pgen.1007290.s006.docx]

**S2 Table**

| **t-Test** | **Site** | **P values** |
| --- | --- | --- |
| Vehicle-no DOPA vs Vehicle-DOPA | Cell Body | 0.323 |
| YM 1000 nM-no DOPA vs YM 1000 nM-DOPA | Cell Body | 0.352 |
| Vehicle-no DOPA vs YM 1000 nM-no DOPA | Cell Body | 0.067 |
| Vehicle-DOPA vs YM 1000 nM-DOPA | Cell Body | 0.022 |
| Vehicle-no DOPA vs Vehicle-DOPA | Dendrite | 0.137 |
| YM 1000 nM-no DOPA vs YM 1000 nM-DOPA | Dendrite | 0.484 |
| Vehicle-no DOPA vs YM 1000 nM-no DOPA | Dendrite | 0.154 |
| Vehicle-DOPA vs YM 1000 nM-DOPA | Dendrite | 0.462 |
