# Supplementary material for: PIKfyve regulates melanosome biogenesis
Source: PLoS Genet. 2018 Mar 27;14(3):e1007290. doi: 10.1371/journal.pgen.1007290 (PMC5889185; doi:10.1371/journal.pgen.1007290)
Supplement: S3 Table — WB = Western Blot. IF = Immunofluorescence. (DOCX) [file pgen.1007290.s007.docx]

**S3 Table**

| **Antibody** | **Host** | **Application** | **Dilution** | **Company** | **Catalog No.** |
| --- | --- | --- | --- | --- | --- |
| Anti-β-Actin | Rabbit | WB | 1:1000 | Cell Signaling | 1385 |
| Anti-Cathepsin D | Rabbit | WB | 1:1000 | Cell Signaling | 2284 |
| Anti-GAPDH | Rabbit | WB | 1:5000 | Cell Signaling | 5174 |
| Anti-HMB45 (PMEL) | Mouse | WB | 1:1000 | Thermo Scientific | MA5-13232 |
| Anti-Melan-A (MART1) | Mouse | IF | 1:2500 | Santa Cruz | Sc-20032 |
| Anti-Tyrosinase (TYR) | Mouse | WB | 1:3000 | Abcam | ab738 |
| Anti-TYRP1 | Mouse | IF | 1:5000 | Abcam | ab3312 |
| anti-rabbit IgG, HRP-linked | Goat | WB | 1:5000 | Cell Signaling | 7074 |
| anti-mouse IgG, HRP-linked | Horse | WB | 1:5000 | Cell Signaling | 7076 |
