# Supplementary material for: PIKfyve regulates melanosome biogenesis
Source: PLoS Genet. 2018 Mar 27;14(3):e1007290. doi: 10.1371/journal.pgen.1007290 (PMC5889185; doi:10.1371/journal.pgen.1007290)
Supplement: S4 Table — For = Forward primer. Rev = Reverse primer. (DOCX) [file pgen.1007290.s008.docx]

**S4 Table**

| **Primer** | **Sequence 5’ to 3’** | **Animal** | **Application** |
| --- | --- | --- | --- |
| *Cre* For | GGTGTCCAATTTACTGACCGTACA | Mouse | Genotyping |
| *Cre* Rev | CGGATCCGCCGCATAACCAGTG | Mouse | Genotyping |
| *PIKfyve^Flox^*For | GAGAAAGGGGACAGTGTTTGGC | Mouse | Genotyping |
| *PIKfyve^Flox^* Rev | CCAGATCTTGCACTGTAACCACAAACCAC | Mouse | Genotyping |
| *ROSA^mTmG^* For | CTCTGCTGCCTCCTGGCTTCT | Mouse | Genotyping |
| *ROSA WT* Rev | CGAGGCGGATCACAAGCAATA | Mouse | Genotyping |
| *ROSA­^mTmG^* Rev | TCAATGGGCGGGGGTCGTT | Mouse | Genotyping |
